# Amaurosis Chinica

**Published:** 1882-10

**Authors:** 


AMAUROSIS CHINICA.

Three cases of amaurosis by the use of strong doses of quinine are reported. The first, a lady thirty-five years of age, suffered from acute endometritis, and took 80 grains of quinine in three hours. Convulsions set in, hearing disappeared entirely, and the vision was destroyed for five days. The pupils were dilated. Color blindness persisted for nearly one month. The second case was a strong man of thirty-eight years, suffering from pneumonia; he took fifteen grains of quinine in five hours. There was total blindness for three days, and vision was limited for nearly one year. The third case was similar to the second; perception of color appeared much later than that of light. Kirchner has made investigations in animals on this subject, and he found that quinine, as well as pure salicylic acid, causes congestion in the cavity of the tympanum and in the labyrinth, which is frequently followed by hæmorrhage.—*Lo Sperimentale.*

THE editor of the French medical journal "*Le Progrès Médical*" publishes a series of lectures, physiological investigations, etc., which promise to be highly interesting. The first series begins with five lectures of the celebrated physiologist, F. M. Charcot. These lectures are truly scientific, without a particle of those mysterious sentences to be found in former lectures of Charcot. It seems that the French medical schools are reforming at the present time, and that they again aspire to the high position among medical schools which they occupied half a century ago. Prof. Charcot is doubtless on the path of true scientific research, as these lectures on the pathogenic conditions of albuminaria satisfactorily prove, and the editor of this series, Dr. Bourneville, who is also an earnest and active worker, is to be congratulated on these well selected publications. In our next number we will give some extracts from these lectures.

